# Frequency of Risk Factors and Outcome of Hospital-Acquired Acute Kidney Injury

**DOI:** 10.7759/cureus.12001

**Published:** 2020-12-09

**Authors:** Hina Iram, Muhammad Ali, Vinod Kumar, Ayesha Ejaz, Shafique A Solangi, Abdul Manan Junejo, Sagheer Ahmed Solangi, Noor Un Nisa

**Affiliations:** 1 Department of Nephrology, Jinnah Postgraduate Medical Centre, Karachi, PAK; 2 Department of Nephrology, Fazaia Ruth Pfau Medical College, Karachi, PAK; 3 Department of Medicine, Sir Syed College of Medical Sciences for Girls, Karachi, PAK; 4 Department of Medicine, Jinnah Postgraduate Medical Centre, Karachi, PAK; 5 Physiology, Jinnah Sindh Medical University, Karachi, PAK

**Keywords:** sepsis, acute kidney injury, hospitalized, gastroenteritis, mortality

## Abstract

Objective: To determine the frequencies of risk factors and the ultimate outcomes of ccute kidney injury (AKI) among hospitalized patients.

Materials and methodology: This prospective, observational study was carried out from September 15, 2018, to March 14, 2019. All admitted patients, both male and female, with AKI, were included. Those with chronic kidney disease (CKD), small size echogenic kidneys (on ultrasonography, performed on admission), and recent history of urological intervention were excluded from the study. All patients were assessed for etiological factors (sepsis, gastroenteritis, surgical, and obstetrical) and outcome (improved, progression to CKD, or expired).

Results: Out of a total of 230, most patients were aged between 20-50 years with a mean age of 38.99 ± 7.61 years. Males were 144 (62.61%) and females were 86 (37.39%). About 78 (33.91%) patients were hypertensive while 65 (28.26%) were diabetic. The cause of hospital-acquired AKI was found to be sepsis in most (71.73%, n=165) of the cases, followed by gastroenteritis (10.00%, n=23), surgical (9.56%, n=22), and obstetric (8.69%, n=20) causes. When the outcome was assessed, 10 (4.35%) patients expired, 154 (66.96%) improved completely, while 66 (28.69%) progressed to CKD.

Conclusion: This study has shown that sepsis is the most common cause of AKI in patients admitted to the hospital. So we recommend that proper steps should be taken to ensure adequate hospital care for avoiding such outcomes in hospitalized patients, and further decrease mortality.

## Introduction

Acute kidney injury (AKI), recently taking over the previous term acute-renal failure (ARF), is a sudden decline in renal function and represents both structural and functional damage. It is a fairly common condition affecting public health globally. There has been a frequent change in the definition of AKI in the past few years. Based on a consensus, the Acute Dialysis Quality Initiative (ADQI) defined ARF and established a staging criterion called the RIFLE criterion (Risk, Injury, Failure, Loss, and End-stage kidney disease) in 2002. The AKI network came into existence in 2004 and introduced the term ‘Acute Kidney Injury’, reflecting the entire array of ARF. According to the proposed diagnostic criteria for AKI (Kidney Disease Improving Global Outcomes - KDIGO), when there is a sudden decline (within 48 hours) in kidney function (serum creatinine >0.3 mg/dL), or >50% increase in serum creatinine, or decreased urine output of <0.5 mL/kg/hr for >six hours, it is diagnosed as AKI [1-4].

There is an increasing frequency of AKI in hospitalized patients, affecting around 22% of adults, complicating the recovery from their primary disorder [5]. To reduce the incidence and further improve the outcome of AKI, immediate diagnosis, and prompt management are required. The mission of healthcare institutions is to know local epidemiology so that they can establish strategies to prevent in-hospital deaths from AKI [6]. These have already been reported in the available literature, is best known in the septic population, within the intensive (ICU), and postoperative (PCU) care units, among others [7].

AKI can lead to adverse outcomes secondary to multi-organ dysfunction including kidneys progressing to chronic kidney disease (CKD). It has an estimated mortality rate of 21% that increases with the increasing severity of AKI [8]. In a study by Evans et al., risk factors for hospital-acquired AKI were found to be sepsis (86.9%) and gastroenteritis (GE) (19.0%) [9]. While in a local study, Khan et al. found sepsis (16.0%), GE (24.0%), surgical (17.0%), and obstetrical (5.0%) as probable causes of AKI. Regarding outcome in these patients who improved at the end of the study, 72% of the cases were managed conservatively, 22% were temporarily dialyzed, 5% were advised long term dialysis as they progress to CKD, and 13% of the cases expired [10]. Another study by the US Veterans Administration reported that increased mortality risk remains post-treatment and is increased twice at one year when compared to those without AKI [11].

Pakistan, being a developing country, does not have a well-developed health care system throughout the country. AKI is one of those disorders that can be a hurdle for managing the primary disease. Still, not much data is available on AKI as to why it develops and how it progresses. This study aimed to determine the frequencies of risk factors and the ultimate outcomes of AKI among hospitalized patients.

## Materials and methods

This prospective, observational study was carried out at the Nephrology Department, Jinnah Postgraduate Medical Centre, Karachi, Pakistan, from September 15, 2018, to March 14, 2019. The sample size was calculated by using the OpenEpi software version 3.01, which came out to be 227 with a 95% confidence level, 5% margin of error, and frequency of AKI in hospitalized patients as 18% [8]. Legal formalities were done by taking informed consent from all patients and ethical committee approval. Non-probability, consecutive sampling technique was used.

All admitted patients, both male and female, with AKI (identified by KDIGO criteria as described above) were included. Those with CKD, small size echogenic kidneys (on ultrasonography, performed on admission), and recent history of urological intervention were excluded from the study. Those who started on renal replacement therapy (RRT) at any time of the study duration were also excluded.

All patients were assessed for etiological factors (sepsis, GE, surgical, and obstetrical) and outcome (improved or progressed to CKD, or expired). CKD was defined as patients with radiological and biochemical evidence of CKD (estimated glomerular filtration rate less than 60 ml/min/1.73 mt2, persisting for three months or more) but not on RRT [12]. Patients were discharged when asymptomatic (i.e. adequate urine output, normalization of creatinine, no acidosis, no hyper/hypokalemia and volume overload). They were followed-up on telephonic interviews and in the outpatient departments for up to six months.

Statistical analysis was done using Statistical Package for Social Sciences (SPSS) version 22 (IBM Corp., Armonk, NY, USA). Mean and the standard deviation was calculated for age while the gender, comorbid (hypertension, diabetes mellitus), etiological factors, and outcome were presented as frequency and percentage. The chi-square test was applied to the variables post-stratification to see their effect on the outcome. A P-value of ≤0.05 was calculated as significant.

## Results

In this study, it was established that most patients were aged between 20 to 50 years with a mean age of 38.99 ± 7.61 years. Out of 230 patients, males were 144 (62.61%) and females were 86 (37.39%) with M:F of 1.7:1.

When comorbidities were assessed, 78 (33.91%) were hypertensive and 65 (28.26%) were diabetic. The cause of hospital-acquired AKI was found to be sepsis in most (71.73%, n=165) of the cases, followed by GE (10.00%, n=23), surgical (9.56%, n=22), and obstetric (8.69%, n=20) causes.

Regarding the outcome in these patients, mortality was seen in 10 (4.35%) patients, 154 (66.96%) had improved completely, while 66 (28.69%) progressed to CKD. Stratification according to different patient characteristics is given in Table 1, although these associations were statistically nonsignificant.

**Table 1:** Association of patient characteristics with mortality and survival (n=230)

Variable	Total	Completely recovered n (%)	Progressed to chronic n (%)	Expired n (%)	p-value
Gender					0.13
Male	144	100 (69.44)	36 (25.00)	8 (5.55)	
Female	86	53 (61.63)	31 (36.05)	2 (2.32)	
Comorbid					0.06
Diabetes mellitus	65	30 (46.15)	29 (44.61)	6 (9.23)	
Hypertension	78	23 (29.49)	50 (64.1)	5 (6.41)	
Etiology					0.22
Sepsis	165	106 (64.24)	54 (32.72)	5 (3.03)	
Gastroenteritis	23	16 (69.56)	5 (21.74)	2 (8.69)	
Surgical	22	18 (81.81)	2 (9.09)	2 (9.09)	
Obstetrical	20	14 (70.00)	5 (25.00)	1 (5.00)	

## Discussion

The epidemiology of AKI in developing countries differs from that of the developed countries in many ways [2,13-20]. In developed countries, elderly patients predominate, while the younger population is more commonly involved in developing countries [21-22]. Compared to the other part of the world, finding the actual incidence of AKI is very difficult when we search for the data in developing countries. Seedat et al. reported an incidence of 20 cases per million populations per year in South Africa [23]. While in North India, Jha et al. reported an incidence of 6.4/1000 admissions per year [24]. AKI is associated with tremendous consumption of health-care resources, morbidity, and mortality especially in patients admitted to the hospital. The occurrence of kidney failure in patients admitted to hospital for non-renal problems complicates the hospital course with an out-and-out impact on patient outcomes. An increased incidence of sepsis and a lack of well-trained personals to deal with intensive care, hamper the efforts to prevent AKI. The costs of RRT are also prohibitively high in developing countries [25-26]. Sepsis was the dominant cause of AKI in our study (64.24%), with a mortality of 3.03%.

In a prospective study, the incidence of AKI was eight per 1000 admissions, of which 7.8% was hospital-acquired AKI. As far as the etiology is concerned, 87.4% had medical diagnoses (sepsis being the most common at 53.1%), 9.4% had surgical, while 3.2% had obstetric problems. Among sepsis, urosepsis and pneumonia were the most common causes. Nearly 38.2% had a multi-organ failure, 23.3% required dialysis, while mortality was 8.7%. Predictive factors of mortality included anemia, the need for intensive care, and the use of vasopressin drugs. Many of these are potentially preventable with early and proper fluid replacement, appropriate antidotes, effective anti-infective treatment, early referral to nephrologists, and specialized dialysis units [16].

In another prospective observational study, a total of 412 patients (52.6% males and 47.4% females, including 7.5% human immunodeficiency virus [HIV] infected patients) were included, out of which 10.9% had AKI. Hypoperfusion and sepsis, mainly because of infection with the malarial parasite (42.2%), were the commonest causes of AKI. In-hospital mortality was 20.5% and 2.9% with and without AKI, respectively. Around 47.2% with renal disease had persistent kidney injury at the time of discharge from the hospital.

The incidence of AKI in hospitalized adults in Africa was found to be 0.3-1.9%, where 90% were not nosocomial. Most of the patients with AKI had a mean age of 41.3±9.3 years and an M:F ratio of 1.2:1. Iatrogenic causes accounted for 65-80% of cases of AKI, followed by obstetric (5-27%), and surgical (2-24%) causes. In the reported studies, 17-94% of patients needed dialysis while the mortality rate was 11.5-43.5% [27].

Hsu et al. in their retrospective work examined 734,340 in Taiwan, where AKI was detected in 1.68-2% of the hospital discharges among adults with and without preexisting CKD respectively. The incidence of hospital-acquired AKI was 814 per 100,000 admissions. In-hospital mortality (26.07% vs 51.58%) was found to be higher with more severe outcomes in patients who had acquired AKI in the hospital unlike that of community-acquired AKI [28].

In a study on 220 patients, where 86.81% survived, GE was the commonest etiology of AKI in 27.3% of participants reflecting the high incidence of disease in the community, followed by sepsis in 14.1% and pyelonephritis in 12.3%. Among these, sepsis had the highest mortality (n=11, 35.5%), and GE and acute febrile illness had the least mortality (1.7% and 0%), respectively (P < 0.005) [29].

Similarly, this study had a higher proportion of males (62.61%) with M:F of 1.7:1. AKI had sepsis as the most common etiology (71.73%, n=165), followed by GE (10.00%, n=23). Regarding the outcome in these patients, mortality was seen in 10 (4.35%) patients, with 8.69% deaths in patients GE and 9.09% in those of surgical causes.

Our study is limited by the fact that mortality was not assessed after hospital discharge and the duration of hospital and especially ICU stay was not determined. Follow-up was just for six months with no assessment of outcomes later. Data was just from a single dialysis center of a public sector hospital. Lastly, a larger sample collection would have given more statistically significant results.

## Conclusions

This study has shown that sepsis is the most common cause of AKI in patients admitted to the hospital. We recommend that proper steps should be taken to ensure adequate hospital care for avoiding such outcomes in hospitalized patients and further decrease mortality.
